# Biochemical and mechanistic analysis of the cleavage of branched DNA by human ANKLE1

**DOI:** 10.1093/nar/gkad416

**Published:** 2023-05-22

**Authors:** Alasdair D J Freeman, Anne-Cécile Déclais, Timothy J Wilson, David M J Lilley

**Affiliations:** Nucleic Acid Structure Research Group, MSI/WTB Complex, The University of Dundee, Dow Street, Dundee DD1 5EH, UK; Nucleic Acid Structure Research Group, MSI/WTB Complex, The University of Dundee, Dow Street, Dundee DD1 5EH, UK; Nucleic Acid Structure Research Group, MSI/WTB Complex, The University of Dundee, Dow Street, Dundee DD1 5EH, UK; Nucleic Acid Structure Research Group, MSI/WTB Complex, The University of Dundee, Dow Street, Dundee DD1 5EH, UK

## Abstract

ANKLE1 is a nuclease that provides a final opportunity to process unresolved junctions in DNA that would otherwise create chromosomal linkages blocking cell division. It is a GIY-YIG nuclease. We have expressed an active domain of human ANKLE1 containing the GIY-YIG nuclease domain in bacteria, that is monomeric in solution and when bound to a DNA Y-junction, and unilaterally cleaves a cruciform junction. Using an AlphaFold model of the enzyme we identify the key active residues, and show that mutation of each leads to impairment of activity. There are two components in the catalytic mechanism. Cleavage rate is pH dependent, corresponding to a p*K*_a_ of 6.9, suggesting an involvement of the conserved histidine in proton transfer. The reaction rate depends on the nature of the divalent cation, likely bound by glutamate and asparagine side chains, and is log-linear with the metal ion p*K*_a_. We propose that the reaction is subject to general acid-base catalysis, using a combination of tyrosine and histidine acting as general base and water directly coordinated to the metal ion as general acid. The reaction is temperature dependent; activation energy *E*_a_ = 37 kcal mol^−1^, suggesting that cleavage is coupled to opening of DNA in the transition state.

## Graphical Abstract

**Graphical Abstract:**
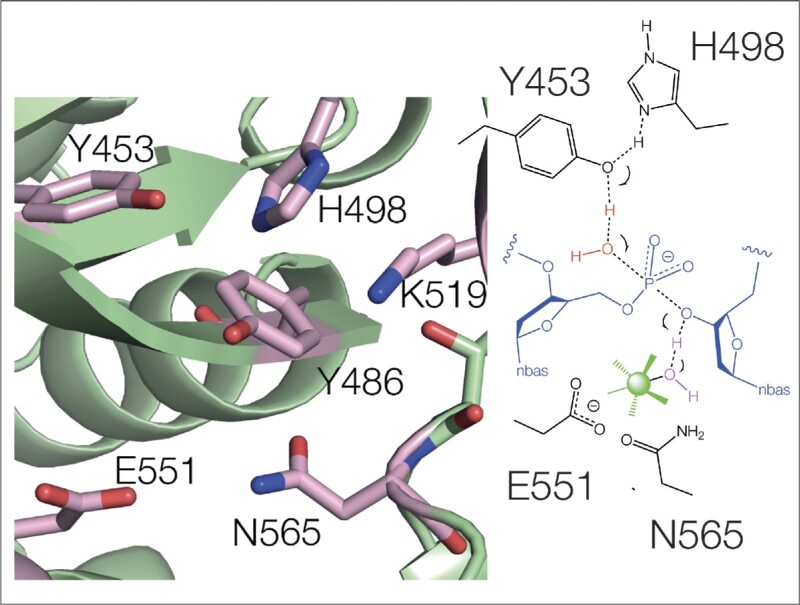

## INTRODUCTION

Recent evidence suggests that LEM-3/ANKLE1 plays an important role in providing a final opportunity to process persistent DNA junctions that have escaped enzymatic processing, and that could lead to DNA bridges linking chromosomes thus blocking cell division. Every physical connection between sister chromatids must be removed before cells can divide. Connections between segregating chromatids exist as chromatin bridges (1–3), or ultrafine bridges (4,5) that are associated with BLM and PICH helicases. LEM-3 was originally identified in a genetic screen for embryonic lethality in *Caenorhabditis elegans* resulting from exposure to ionizing irradiation (6). ANKLE1 is the corresponding ortholog in humans and other metazoans (7).

LEM-3/ANKLE1 protein sequences include predicted GIY-YIG nuclease domains at the C-terminus, and LEM3 was shown to exhibit nucleolytic activity on supercoiled DNA (6). We have recently shown that human ANKLE1 carries out structure-selective nucleolytic cleavage directed towards a variety of branched DNA species (8). These branchpoints would be expected to result from the failure to process recombination intermediates fully, or perhaps replication intermediates, and such structures could result in the formation of inter-chromosomal linkages. A number of lines of evidence suggest that LEM-3/ANKLE1 might be responsible for processing DNA bridges during anaphase:

1. LEM-3 is known to interact genetically with the Holliday junction-processing enzymes MUS81, SLX1 and SLX 4, with embryonic lethality resulting in lem-3 double mutants (3,9)
2. Cytological experiments have revealed that LEM-3 accumulates at the cellular mid-body where the two daughter cells abscise from each other (10)
3. In lem-3 mutants treated with DNA damaging agents, and when DNA replication or chromosome decondensation are compromised then chromosomal bridges persist (3).

Thus ANKLE1 has very much the substrate specificity expected for a nuclease whose role is to catch unprocessed or partially processed junctions late in cell division (8), and its ortholog LEM3 has been shown to be located just at the point in the dividing cells consistent with that role (10). ANKLE1 has thus been described as the ‘enzyme of last resort’. Despite clear indications of the probable importance of human ANKLE1, very little is known about how the enzyme operates as a nuclease. Like the SLX1 component of the multi-component SLX1–SLX4–MUS81–EME1 resolution complex it appears to be a GIY-YIG nuclease; these are relatively rare in eukaryotes. There have been a number of crystallographic studies of different GIY-YIG nucleases (11–18), but very little mechanistic analysis. Given its likely significance in cell division it is a potential therapeutic target, and therefore it is of both intellectual and practical importance to learn more concerning the mechanism of ANKLE1 nuclease. Moreover aspects of catalytic mechanism learned for ANKLE1 can likely provide insight into the mechanism of other GIY-YIG nucleases, including SLX1.

## MATERIALS AND METHODS

### Bacterial cloning and mutagenesis of human ANKLE1

N-terminal deletions of human ANKLE1 were generated by PCR of the coding region of isoform 1 of human Ankle1 (NCBI Reference Sequence: NP_689576), cloned into pGEX6P, using the Q5 Site-Directed mutagenesis kit (New England Biolab E0554S). DNA primers were chosen to delete sequences between the N-terminus and chosen positions (Supplementary Table S1). PCR products were transformed into *E. coli* DH5α and single colonies were grown in LB broth containing 100 mg/l carbenicillin and 50 μM IPTG. After 16 h, induced protein production was analysed by SDS PAGE from an aliquot of the culture medium, while the remaining culture was used to purify plasmid DNA by mini-prep (Qiagen). A deleted form beginning at methionine 331 (ANKLE1_331–615_) was found to be fully active, and used in all the experiments described in this work. This was found to contain a mutation R492H that facilitated growth in *E. coli*. Site directed mutation of active site residues were performed by PCR using the same Q5 site directed mutagenesis kit and the appropriate primers (Supplementary Table S1). All DNA constructs were verified by DNA sequencing.

### Expression and purification of ANKLE1_331–615_ R492H in *E. coli*

Arctic Express *E. coli* were transformed with pGEX6P-ANKLE1_331–615_ R492H and its mutants and grown in LB supplemented with 100 mg/l carbenicillin and 20 mg/l gentamicin at 37°C until the OD_600_ of the culture reached 0.6. The temperature of the culture was lowered to 12°C. When the absorbance of the culture at 600 nm was between 1.0 and 1.5, ANKLE1_331–615_ R492H production was induced by the addition of IPTG to a final concentration of 200 μM and incubated for 24 h. Bacterial cells were harvested by centrifugation at 7500 g for 15 min at 4°C. The cell pellet was resuspended in 40 ml/l of cell culture of 25 mM HEPES (pH 7.5), 1M NaCl, 5% glycerol, 0.5 mg/ml lysozyme and 1 tablet of complete protease inhibitors (Roche) and incubated at room temperature for 15 min. Triton X100 was added to a final concentration of 0.2% (w/v) and the extract was then sonicated on ice at 30 W for 1.5 min using 10 s pulses separated by 30 s pauses using a FB705 sonicator (Fischer Scientific). The cellular extract was clarified by centrifugation at 20 000 g for 30 min at 4°C (Beckman rotor JA25.50). The supernatant was mixed with glutathione sepharose 4B beads (GE healthcare), which were preequilibrated with 25 mM HEPES (pH 7.5), 1 M NaCl, 5% glycerol. The beads were then washed with the same buffer. GST-ANKLE1_331–615_ R492H was eluted as a fusion protein by incubating the beads with 25 mM HEPES (pH 7.5), 50 mM NaCl, 5% glycerol and 20 mM glutathione. Alternatively, GST-ANKLE1_331–615_ R492H was cleaved with human 3C protease while bound to the glutathione column overnight in 25 mM HEPES (pH 7.5), 150 mM NaCl, 5% glycerol. GST fusion or non-fusion ANKLE1_331–615_ R492H were further purified by chromatography on Fractogel SO^3_−_^ (Merck) in 25 mM HEPES (pH 7.5), 5% glycerol with a linear NaCl gradient from 50 mM to 1 M, followed by purification on Mono S 5/50 GL (GE Healthcare) in 25 mM HEPES (pH 7.5), 10% glycerol, 0.5 mM EDTA with a linear NaCl gradient from 50 mM to 1 M.

Protein concentration was measured by UV spectrophotometry using an extinction coefficient of 36 500 M^−1^ cm^−1^ for ANKLE1_331–615_ R492H or 79 600 M^−1^ cm^−1^ for GST-ANKLE1_331–615_ R492H and stored at –70°C.

### Denaturing gel electrophoresis of proteins in SDS-containing buffer

ANKLE1_331–615_ R492H purity was analysed by polyacrylamide gel electrophoresis in denaturing condition. Protein samples were subjected to electrophoresis in 10% polyacrylamide Bis–Tris NuPAGE (Invitrogen) in 50 mM MOPS, 50 mM Tris (pH 7.7), 3.47 mM SDS, 1 mM EDTA. Protein bands were detected using Instant Coomasie Protein Stain (Abcam) and electrophoretic migration was compared to standard proteins (PageRuler Plus Prestained Protein Ladder, Thermo Scientific).

### Construction of DNA splayed Y-junctions

Oligonucleotides were purchased from Sigma-Aldrich.

Splayed Y-junction was prepared by the hybridization of r- and x-strands (Supplementary Table S1). Where required, strands were radioactively-[5′-^32^P]-labelled by polynucleotide kinase (Thermo Scientific). 2 μM of oligonucleotide was incubated with 0.17 μM [γ-^32^P]-ATP at 37°C in 50 mM Tris (pH 7.6), 10mM MgCl_2_, 5 mM DTT, 0.1mM spermidine for 30 min. 50 mM EDTA was added to terminate the kinase reaction before the addition of the other oligonucleotide in order to generate the splayed Y-junction substrate. Oligonucleotides were hybridized to generate splayed Y-junctions by slow cooling from 80°C to 4°C. The substrate was purified by electrophoresis in 8% (29:1) polyacrylamide gels in 90 mM Tris–boric acid (pH 8.3), 2 mM EDTA.

### Analysis of the multimeric state of ANKLE1_331–615_ R492H by gel filtration

ANKLE1_331–615_ R492H was subjected to gel filtration on Superdex 75 (10/300) at a flow rate of 0.4 ml min^−1^ in 25 mM HEPES (pH 7.5), 0.1 M NaCl, 0.1 mM EDTA, 10% glycerol. Elution of ANKLE1_331–615_ R492H was compared to that of carbonic anhydrase (C-7025, Sigma) with an estimated molecular mass of 29 000 Da

### Measurement of binding affinity by fluorescence anisotropy

The r-strand was synthesized with fluorescein attached at the 3′ terminus, using a 6-fluorescein CPG column (Glen Research 20-2961). The splayed Y-junction was generated by hybridization with the h-strand of J3. Fluorescent anisotropy measurements were performed with a SLM Aminco 8100 fluorescence spectrometer. The excitation wavelength was selected using an interference filter of 482.5 ± 31 (fwhm) nm, and the emission was selected using a monochrometer set at 530 nm with slits set at 8 (fwhm) nm. Fluorescence was measured at 20°C using 100 nM splayed Y-junction in 20 mM HEPES–NaOH (pH 7.0 or 8.0), 50 mM KCl, 2 mM MnCl_2_, 0.1 mg/ml BSA. ANKLE1_331–615_ R492H Y453F was added to the indicated concentrations. Fluorescence anisotropy (*r*) was measured as :


$$\begin{equation*}r\ = \frac{{{I}_{{\rm VV}} - G{I}_{{\rm VH}}}}{{{I}_{{\rm VV}} + 2G{I}_{{\rm VH}}}}\ \end{equation*}$$

where *I*_VV_ and *I*_VH_ are fluorescence intensity with the polarizers parallel and crossed respectively, and *G* is the ratio of detector sensitivity between vertical and horizontal polarization, and was measured for each determination in these studies.

The data were fitted to the following binding model :


$$\begin{equation*}r\ = {r}_0\ + \Delta {r}_\cdot \frac{{{K}_d + \left[ {Ank} \right] + \left[ {Y} \right] - {{\left( {{{\left( {{K}_d + \left[ {Ank} \right] + \left[ {Y} \right]} \right)}}^2 - 4\left[ {Ank} \right]\cdot\left[ {Y} \right]} \right)}}^{1/2}}}{{2\cdot\left[ {Y} \right]}}\end{equation*}$$

where *r*_0_ is the anisotropy in the absence of protein, Δ*r* is the change in anisotropy over the full range of protein concentration, *K*_d_ is the dissociation constant for ANKLE1 binding to the splayed Y-junction, [*Ank*] and [*Y*] are the ANKLE1_331–615_ R492H and splayed Y-junction DNA concentrations respectively.

### Structural prediction by AlphaFold

The structure of Ankle1 was predicted by the software *AlphaFold* (model Q8NAG6). The overall confidence of the model for the nuclease domain (residue 448–566) of Ankle1 was mostly very high (64%) and the positioning of the backbone of the residues of the mutants in this study were either very high (Y453, Y486, K519 and E551) or confident (H498 and N565).

### Kinetic analysis of ANKLE1 cleavage reactions

5 nM radioactively-[5′-^32^P] x-strand labelled splayed Y-junction DNA was preincubated with 2 μM ANKLE1_331–615_ R492H (or active site mutant) in 20 mM cacodylate (pH 6.5), 50 mM KCl, 0.1 mg/ml BSA at 37°C. After 3 min incubation, MnCl_2_ (or other metal chlorides as indicated in the text) was added to a final concentration of 10 mM to initiate the cleavage reaction. Aliquots were removed at chosen times and the reaction terminated by addition of an equal volume of 25 mM EDTA, 95% formamide. DNA substrate and products were separated by electrophoresis in a 20% (19:1) polyacrylamide gel electrophoresed in 90 mM Tris–boric acid (pH 8.3), 2 mM EDTA, 8 M urea. Radioactivity was detected by exposure to a storage phosphor screen, and quantified using a BAS-1500 phosphorimager (Fuji), or a Typhoon FLA 9500 fluorimager (GE Healthcare). Reaction progress was fitted by non-linear regression analysis to single exponential functions using KaleidaGraph (Abelbeck Software).

For the measurement of cleavage rate as a function of temperature, conditions were identical apart from the incubation temperature.

For the study of the effect of flap lengths, a lower enzyme concentration was used (200 nM) and the products were resolved on a 15% (19:1) polyacrylamide sequencing gel, that was dried before exposure. All other conditions were as above.

For measurement of cleavage rate as a function of pH the following buffers were used for the indicated pH ranges : MES (pH 5.51, 6.01 and 6.39), MOPS (pH 6.5 and 7.2) and HEPES (pH 6.86, 7.58 and 7.8). pH was measured for the reaction buffer (20 mM buffer, 50 mM KCl and 10 mM MnCl_2_) using an Accumet AB150 pH meter (Fischer Scientific) at 31.6°C.

### Cleavage of cruciform structures in negatively supercoiled plasmid DNA

The plasmid pHRX3 (19) was used in these experiments as it contains the sequence of x strand of J3. Negatively supercoiled pHRX3 was purified from *E. coli* DH5α with two rounds of CsCl density gradient centrifugation. Cleavage was carried out similarly to cleavage of splayed Y-junctions; 5 nM pHRX3 was preincubated with 2 μM ANKLE1_331–615_ R492H in 20 mM MES (pH 5.5), 50 mM KCl, 0.1 mg/ml BSA at 31.6°C. After 3 min incubation MnCl_2_ was added to a final concentration of 10 mM to initiate the cleavage reaction. Aliquots were removed at chosen times and the reaction terminated by addition of 50 mM EDTA final concentration. 20 μg Proteinase K (Thermo Scientific) was added and incubated a further 1 h at 37°C. DNA was separated by electrophoresis in a 1% agarose gel in 90 mM Tris-boric acid (pH 8.3), 2 mM EDTA. DNA was stained with SYBR Safe and quantified by fluorescence on a Typhoon FLA 9500 fluorimager (GE Healthcare). Reaction progress of cleavage of supercoiled DNA was fitted to two exponential functions :


$$\begin{equation*}{\rm{\ }}{f}_{cl} = \left( {\left( {{k}_2.{e}^{ - {k}_1t}} \right) - \left( {{k}_1.{e}^{ - {k}_2t}} \right)} \right)/\left( {{k}_2 - {k}_1} \right)\ \end{equation*}$$

where *f*_cl_ is the fraction of supercoiled DNA cleaved at time *t*, and *k*_1_ and *k*_2_ are the two rate constants.

### Permanganate probing of DNA structure

100 nM splayed Y-junction DNA radioactively-[5′-^32^P]-labelled on the indicated strand was pre-incubated at room temperature for 5 min in the presence of 0, 20 nM, 200 nM or 2 μM ANKLE1_331–615_ R492H in 10 mM cacodylate (pH 6.5), 50 mM KCl, 50 μg/ml calf thymus DNA and either 1 mM EDTA (for the active enzyme) or 10 mM MgCl_2_ (for the Y453F mutant). The 20 μl reactions were initiated by adding 2 μl of a 12.5 mM KMnO_4_ solution and terminated by addition of 1.5 μl of β-mercaptoethanol after 1 min. Reaction products were precipitated by addition of 1/10 volume of 3 M sodium acetate and 3 volumes of ethanol, resuspended in 100 μl of 1 M piperidine and incubated at 95°C for 30 min. Samples were dehydrated by addition of *n*-butanol and removal of the solvent phase, followed by vacuum desiccation. Dried samples were resuspended in formamide and analysed on denaturing 15% polyacrylamide sequencing gels. These were dried, exposed to storage phosphor screens and visualised with a Typhoon FLA 9500 fluorimager (GE Healthcare). Purine sequence markers were generated by subjecting the same substrates to reaction with formic acid (20) followed by piperidine cleavage as described above.

## RESULTS

### Expression of human ANKLE1 in bacteria

**Figure 1.**
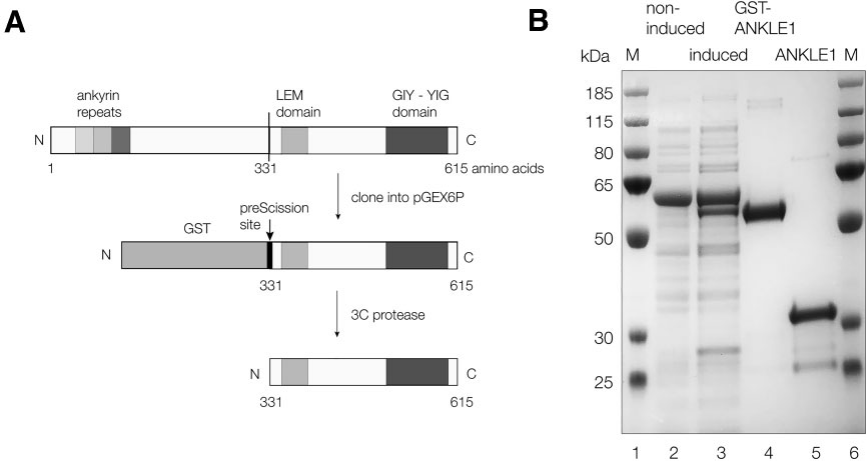
Expression of human ANKLE1 in *Escherichia coli*. (**A**) Scheme showing the cloning and expression of the C-terminal section of hANKLE1. The full-length protein has N-terminal ankyrin repeats, a central LEM domain and the GIY-YIG putative active center at the C-terminus. The C-terminal section beginning at methionine 331 was generated by PCR and fused at its N-terminus to GST to act as an affinity tag. After expression and purification on glutathione Sepharose 4B beads, the fusion protein was cleaved with 3C protease to release the hANKLE1_331–615_ domain used in these studies. This protein had acquired a R492H mutation during genetic manipulation allowing better expression. (**B**) Analysis of the bacterial expression of hANKLE1_331–615_ by denaturing gel electrophoresis in a 10% polyacrylamide Bis-Tris NuPage gel in SDS. Tracks 2 and 3 contain 10 μl aliquots of bacterial culture non-induced and induced by IPTG respectively. The major band is a chaperone protein that co-purifies with GST-ANKLE1. Track 4 contains purified GST-hANKLE1_331–615_R492H fusion protein, and track 5 contains hANKLE1_331–615_R492H after cleavage with Prescission protease and affinity purification. Tracks 1 and 6 contain protein molecular mass markers (sizes in kDa written on the left).

While we have previously expressed human ANKLE1 in insect cells (8) it would clearly facilitate analysis to express active enzyme in bacteria, where site-specific mutants could be easily generated. N-terminal deletions of human ANKLE1 were generated by PCR of the coding region of isoform 1 of human ANKLE1 (Figure 1A). We found that the C-terminal fragment beginning at methionine 331 (see the protein sequence in Supplementary Figure S1) was fully active, so this fragment (termed ANKLE1_331–615_) was used in all subsequent analysis. Sequencing revealed that this contains a R492H mutation that facilitated growth in *E. coli* without preventing activity. This mutation is present in all constructs used in this work. hANKLE1_331–615_R492H was expressed as an N-terminal fusion with GST, allowing purification on glutathione sepharose 4B beads. The fusion protein was cleaved while bound to the glutathione column with human 3C protease to release the isolated hANKLE1_331–615_R492H fragment. This was further purified by chromatography on two sequential ion-exchange columns. The purity of the final hANKLE1_331–615_R492H product was demonstrated by polyacrylamide gel electrophoresis under denaturing conditions (Figure 1B).

### Human ANKLE1_331–615_R492H expressed in bacteria is active

The pure human hANKLE1_331–615_R492H expressed in bacteria is highly active. The pattern of cleavage on a splayed Y-junction DNA (Supplementary Figure S2) shows cleavage from 3 nt into the duplex from the branchpoint, progressively moving down the duplex region. This is exactly what was observed using the form of human ANKLE1 expressed in insect cells (8). hANKLE1_331–615_R492H cleaves the splayed Y-junction DNA with a rate of 52 ± 1.7 × 10^−3^ s^−1^ under our standard conditions in Mn^2+^ ions at 37°C. While the standard substrate has two single-stranded regions of 25 nt in length, these could be substantially shortened (either the 3′ or the 5′ strand, or both symmetrically) with retention of activity (Supplementary Figure S2).

### ANKLE1_331–615_R492H is monomeric in free solution

**Figure 2.**
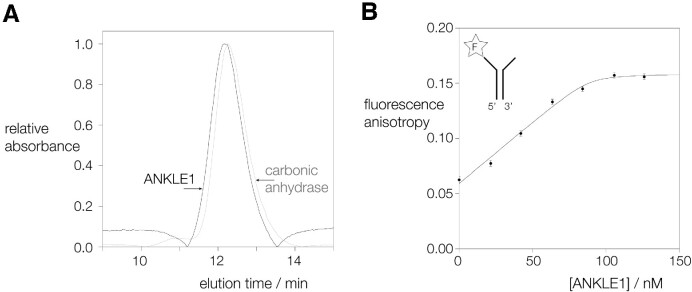
Human ANKLE1 is monomeric in free solution, and binds to a junction as a monomer with nanomolar affinity. (**A**) Estimation of the multimeric state of hANKLE1_331–615_R492H in solution by gel filtration. hANKLE1_331–615_R492H and carbonic anhydrase (used here as a molecular mass standard) were applied separately to a Superdex 75 (10/300) column in 20 mM HEPES (pH 7.5), 100 mM NaCl, 10% glycerol, 1mM EDTA, and elution monitored by absorbance at 280 nm. The elution profiles reveal that hANKLE1_331–615_R492H elutes at a closely similar point to carbonic anhydrase (molecular mass 29 kDa). Since the calculated molecular mass of hANKLE1_331–615_R492H is 33 kDa this indicates that it is predominantly monomeric in solution. (**B**) Binding of hANKLE1_331–615_R492H Y453F to a splayed Y-junction analysed by fluorescence anisotropy. 100 nM splayed Y-junction with fluorescein attached at the 3′ end of the single-stranded section (see insert) was titrated with hANKLE1_331–615_R492H and fluorescence anisotropy measured with excitation at 482.5 nm and emission at 530 nm. Fluorescence was measured in 20 mM HEPES–NaOH (pH 7.0), 50 mM KCl, 2 mM MnCl_2_, 0.1 mg/ml BSA at 20°C. The data are fitted to the binding isotherm described in the text. Note that the plateau value of the anisotropy is reached at 100 nM protein, showing that the stoichiometry is 1:1 protein:DNA.

Holliday junction-resolving enzymes in general bind to four-way DNA junctions in dimeric form, and introduce symmetrically-paired cleavages within the lifetime of the DNA-protein complex (21–24). While ANKLE1 will cleave four-way junctions, it does so more slowly than splayed Y-junction DNA (8), its best substrate. It therefore seemed probable that ANKLE1 acts in monomeric form. We analyzed the state of multimerization of hANKLE1_331–615_R492H using gel filtration. Our purified protein was applied to a Superdex 75 (10/300) column and its elution profile measured by light absorption (Figure 2A). In parallel a sample of carbonic anhydrase (molecular mass = 29000 Da) was applied to the same column. The elution profiles of the two proteins are closely similar. Since the calculated molecular mass for a monomer of ANKLE1_331–615_ is 33000 Da we conclude that hANKLE1_331–615_R492H is monomeric in solution.

### ANKLE1_331–615_R492H binds to a junction in monomeric form, with nanomolar affinity

We have analysed the binding of catalytically-inactive hANKLE1_331–615_R492H Y453F to splayed Y-junction DNA by following the fluorescence anisotropy of fluorescein attached to the 3′ terminus of the single-stranded section. 100 nM of fluorescent splayed Y-junction was titrated with increasing concentrations of ANKLE1_331–615_R492H and the values of anisotropy plotted in Figure 2B. The anisotropy of the fluorophore attached to free DNA is 0.06, indicating that the fluorescein is very mobile. On addition of hANKLE1_331–615_R492H the anisotropy increases showing that mobility of the fluorophore becomes restricted on protein binding. There is a linear increase in anisotropy up to a plateau; this is characteristic of stoichiometric binding, where the concentration of DNA is significantly greater than the *K*_d_. The plateau value of the anisotropy of 0.15 is reached at 100 nM protein, i.e. the same as the concentration of the splayed Y-junction DNA, showing that the stoichiometry is 1:1 protein:DNA. The data can be fitted to a simple binding isotherm. Although the binding affinity cannot be measured with high precision, simulation of the binding isotherms (Supplementary Figure S3A) indicates that the dissociation constant *K*_d_ is close to 1 nM. These data indicate that a single molecule of hANKLE1_331–615_R492H binds to the splayed Y-junction with high affinity.

The titration was repeated at the higher pH value of 8. Under these conditions only a very small change in anisotropy was measured (Supplementary Figure S3B), indicating that very little binding occurs at this pH.

### ANKLE1_331–615_R492H makes a single incision into a cruciform four-way junction

**Figure 3.**
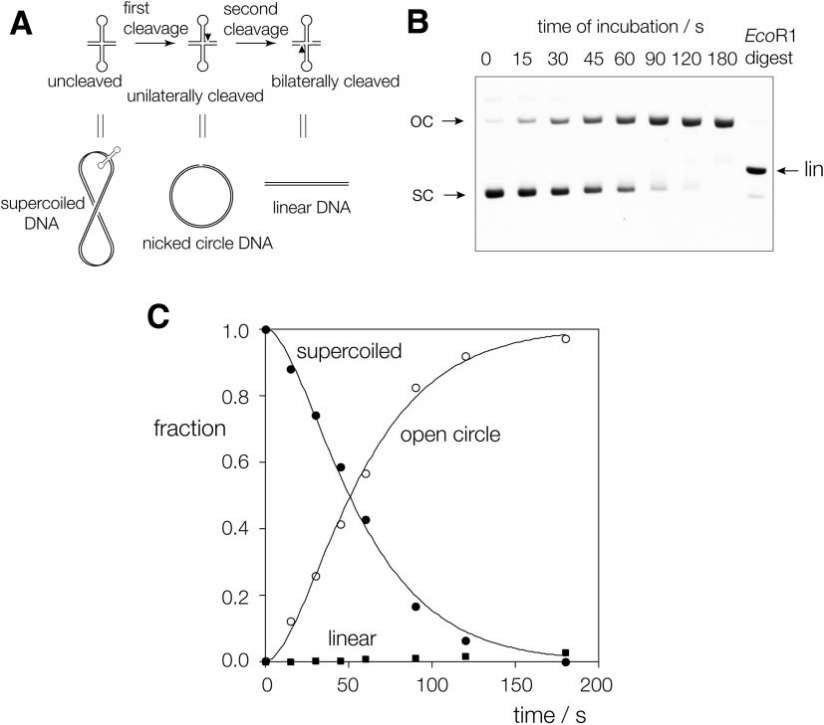
Analysis of unilateral vs bilateral cleavage of a four-way junction by hANKLE1 using a supercoil-stabilized cruciform structure. (**A**) The principle of the use of a cruciform-containing supercoiled plasmid to distinguish unilateral and bilateral cleavage of the four-way junction. Unilateral cleavage results in the formation of nicked circular DNA, while bilateral cleavage would result in linearization. Supercoiled, nicked circular and linear DNA may be separated by electrophoresis in an agarose gel. (**B**) 5 nM of pHRX3 DNA was incubated with 2 μM hANKLE1_331–615_R492H, aliquots removed at indicated times and the different DNA species separated by electrophoresis in a 1% agarose gel. These were stained using SYBR Safe and quantified by fluorimaging. The final track contains plasmid linearised by cleavage at its single *Eco*RI site to show the migration position of linear DNA. The gel shows that cleavage with hANKLE1_331–615_R492H results in complete conversion to open circular DNA, with no formation of linear product, i.e. only unilateral cleavage occurs. (**C**) The quantified data from (B) are plotted as a function of time, and the disappearance of supercoiled DNA (closed circles) and the formation of open circular product (open circles) fitted to double exponential functions (lines). The data for the linear DNA are plotted as closed squares.

The gel filtration data indicate that hANKLE1_331–615_R492H is monomeric in free solution, and the binding experiments show that it binds to splayed Y-junction in monomeric form. We would therefore expect that it cleaves only one strand in a DNA branchpoint. The best way in which to distinguish unilateral and bilateral cleavage of a four-way junction is to study the cleavage of a cruciform in a negatively supercoiled plasmid (21–23); the cruciform comprises a four-way junction connecting two stem-loops and the two arms of the plasmid. The principle is shown in Figure 3A. If an enzyme introduces a single cleavage this releases the supercoiling and the cruciform is reabsorbed so that there is no substrate remaining to be cleaved a second time. In that situation the DNA is cleaved on one strand, leaving an open circular DNA product. However, two cleavages can be made if they occur simultaneously, or within the lifetime of the enzyme-DNA complex. In that case the product will be linear plasmid. Since supercoiled, open circular and linear forms of the plasmid DNA are readily separated by agarose gel electrophoresis this provides a straightforward test distinguishing between unilateral and bilateral cleavage.

A time course of cleavage of a supercoiled cruciform-carrying plasmid by hANKLE1_331–615_R492H is shown in Figure 3B, and the results quantified and plotted in Figure 3C. Over a 2 min. incubation the nuclease ANKLE1_331–615_R492H converts supercoiled plasmid into open circular, with no linear product visible. This demonstrates that hANKLE1_331–615_R492H introduces a unilateral cleavage into the cruciform junction, consistent with the enzyme acting as a monomer that cleaves one strand only. This is in marked contrast with GEN1 for example, where the open circular form is generated as a transient intermediate and linear DNA is the eventual product, showing that like other junction-resolving enzymes it acts as a dimer making bilateral cleavages (24). Reaction progress was best fitted using two exponential functions, indicating a brief lag phase prior to cleavage of the DNA.

### The active center of ANKLE1

**Figure 4.**
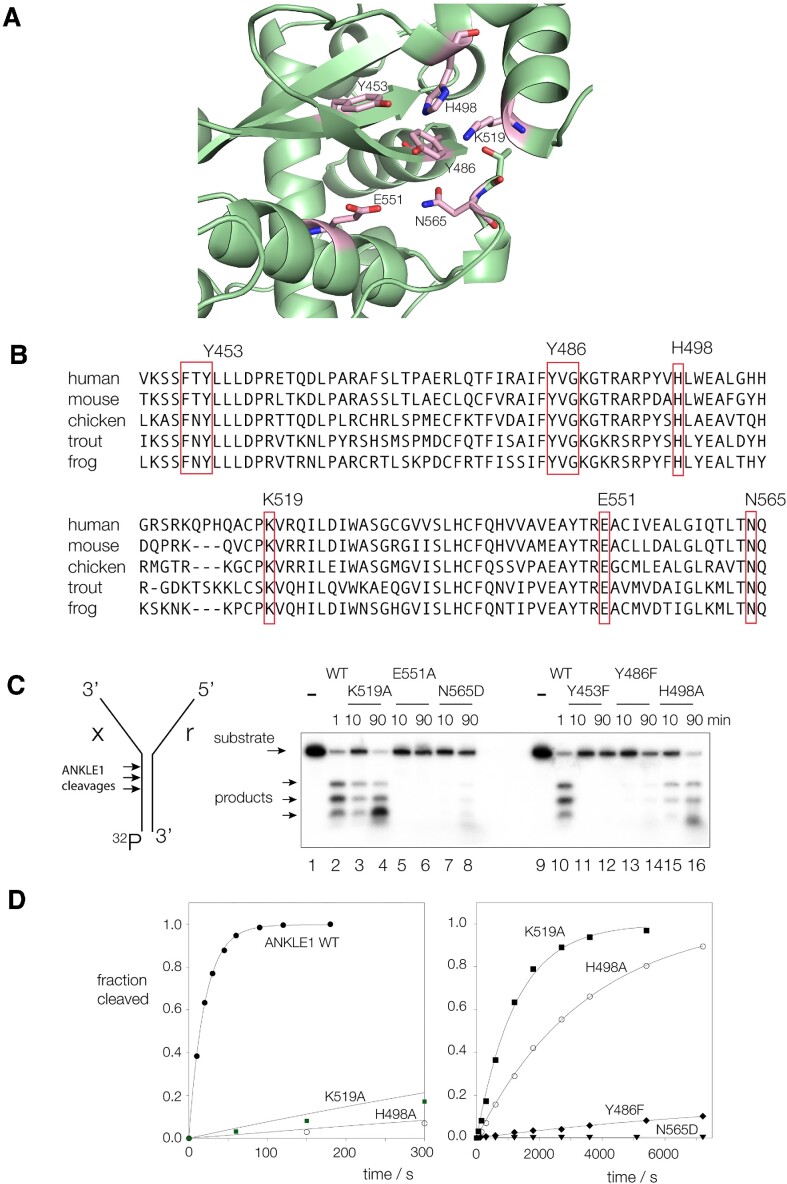
The predicted active site of hANKLE1, and analysis by mutagenesis. (**A**) The GIY-YIG domain of hANKLE1 predicted by AlphaFold (entry Q8NAG6). Putative active site residues are indicated in stick form, colored magenta. (**B**) Alignment of the GIY YIG nuclease domain between ANKLE1 from human, mouse, chicken, trout and frog. The putative active site residues are boxed and are seen to be fully conserved. (**C**) Effect of point mutation of putative active site residues on cleavage activity. Radioactively-[^32^P]-labeled splayed Y-junction DNA was incubated with natural-sequence and point-mutated hANKLE1_331–615_R492H variants for the indicated times. DNA and enzymes were incubated in 20 mM cacodylate (pH 6.5), 50 mM KCl, 0.1 mg/ml BSA at 37°C, and reactions initiated by the addition of MnCl_2_ at a final concentration of 10 mM. Product formation was analysed by electrophoresis in 20% polyacrylamide gels under denaturing conditions, and visualized and quantified by phosphorimaging. (**D**) Reaction progress plotted for junction cleavage by hANKLE1_331–615_R492H and point mutants. The data have been fitted to single exponential functions. Rates are shown in Table 1.

At the present time there is no crystal structure of ANKLE1 or any ortholog including LEM3. However, the structure of human ANKLE1 has been modelled using AlphaFold (25), and was downloaded from Uniprot (26) as model Q8NAG6. The putative active center of the enzyme is shown in Figure 4A. The predicted secondary structure conforms closely to that of other GIY-YIG nucleases for which structures have been determined by X-ray crystallography, including I-TevI (11), UvrC (12), R.*Eco*29kl (13) and SLX1 (15,18). Each comprises three antiparallel β-strands surrounded by α-helices. An alignment of putative active site sequences in vertebrates is shown in Figure 4B; all the expected conserved critical amino acids are present in human ANKLE1 and 100% conserved. These are shown on the AlphaFold prediction in stick form. They comprise the tyrosine residues of the GIY-YIG motif (Y453 and Y486), together with a histidine (H498), glutamate (E551), lysine (K519) and asparagine (N565). Each occurs in the position equivalent to that of the nucleases of known structure.

Each putative active center residue has been mutated, and the rate of cleavage of the splayed Y-junction substrate measured (Figure 4C, D and Table 1) in the presence of Mn^2+^ ions. Mutations at each amino acid led to enzymes that were impaired in cleavage activity, from a 60-fold reduction (K519A) to no detectable activity (Y453F and E551A). The results are consistent with these conserved amino acid side chains playing important roles in the catalytic mechanism of the nuclease. The proximity of Y453, Y486 and H498 in the modelled active center and their sensitivity to mutation suggests a potential role in general acid-base catalysis, as suggested for UvrC (12) and R.*Eco*29kl (13). Given the probable role of a divalent metal ion in catalysis it is also very possible that E551 and N565 coordinate a metal ion, discussed below.

**Table 1.** Rates of cleavage by ANKLE1_331–615_ R492H (denoted here as ANK) and active-site mutants on the splayed Y-junction substrate. Radioactively-[^32^P]-labelled splayed Y-junction DNA was incubated with hANKLE1_331–615_R492H and point-mutated variants in 20 mM cacodylate (pH 6.5), 50 mM KCl, 0.1 mg/ml BSA at 37°C, and reactions initiated by the addition of the indicated divalent metal salt to a final concentration of 10 mM. Rates were measured by fitting reaction progress to single exponential functions. Rates were measured in triplicate, and the average and standard deviations reported. We estimate the minimum detectable rate is 10^−6^ s^−1^

ANKLE1	Ion	Rate (s^−1^)	Fold reduced
ANK	Mn^2+^	5.2 ± 0.2 × 10^−2^	(1)
ANK	Mg^2+^	1.5 ± 0.1 × 10^−2^	3.5
ANK	Co^2+^	0.27 ± 0.1^a^	(0.19)
ANK	Ca^2+^	<10^−6^	>50 000
Y453F	Mn^2+^	<10^−6^	>50 000
Y486F	Mn^2+^	1.5 ± 0.1 × 10^−5^	3500
H498A	Mn^2+^	3.5 ± 0.2 × 10^−4^	150
K519A	Mn^2+^	8.5 ± 0.2 × 10^−4^	61
E551A	Mn^2+^	<10^−6^	>50 000
N565D	Mn^2+^	<10^−6^	>50 000

(a) In the presence of Co^2+^ ions the reaction was biphasic; the rate shown is the faster initial phase.

### The dependence of ANKLE1_331–615_R492H cleavage on the nature of the metal ion

**Figure 5.**
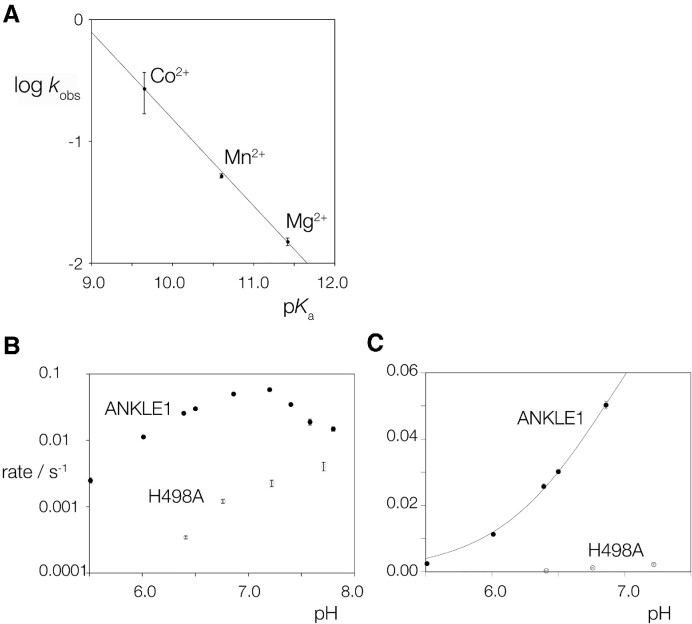
The dependence of the rate of cleavage of splayed Y-junction DNA by hANKLE1_331–615_R492H on the nature of the divalent cation, and on pH. (**A**) Cleavage in the presence of Co^2+^, Mn^2+^ or Mg^2+^ as the divalent cation. Rate of cleavage is plotted on a logarithmic scale as a function of the p*K*_a_ of the divalent metal ions present. The data have been fitted to a straight line. Error bars represent one standard deviation. (**B** and **C**) The pH dependence of the rate of cleavage of splayed Y-junction DNA by hANKLE1_331–615_R492H and hANKLE1_331–615_R492H H498A in the presence of Mn^2+^ ions. The cleavage rate has been measured at a series of pH values between 5.5 and 7.8 under otherwise standard conditions at 31.6°C. In (**B**), the rate of cleavage plotted on a logarithmic scale as a function of pH over the complete range for hANKLE1_331–615_R492H (closed circle symbols) and hANKLE1_331–615_R492H H498A (open circle symbols). The lowering of rate above pH 7 cannot be fitted to a simple ionization process, and measurements of binding indicate that affinity becomes lower at these pH values (Supplementary Figure S3). In (**C**), the rate of cleavage is plotted on a linear scale as a function of pH over the range 5.5 to 7.0. Rates have been measured in triplicate, and the error bars are one standard deviation. In most cases these are smaller than the symbols. These data for hANKLE1_331–615_R492H have been fitted to a single ionization indicated by the line. From this a p*K*_a_ of 6.9 has been calculated.

Our standard conditions for the study of the cleavage of DNA junctions by hANKLE1_331–615_R492H include Mn^2+^ ions. We have explored the rate of cleavage on the splayed Y-junction in different divalent metal ions. The activity is 3.5-fold lower in the presence of Mg^2+^ ions, but the initial rate of cleavage is 5.2 fold faster in Co^2+^ ions (Table 1). The enzyme is essentially inactive in the presence of Ca^2+^ ions. Thus the rate follows the order Co^2+^ > Mn^2+^ > Mg^2+^ >> Ca^2+^. While the low activity in Ca^2+^ ions is likely a result of differences in size and coordination, cleavage activity is log-linear with the p*K*_a_ of the other three ions (Figure 5A). These data are consistent with proton transfer by metal ion-bound water molecules in the transition state, or potentially with the metal ion acting as a Lewis acid. As we discuss below, it is likely that a divalent metal ion is bound into the active site to act as a general acid in the hydrolysis of the phosphodiester bond.

### The dependence of ANKLE1_331–615_R492H cleavage rate upon pH

Given the possible involvement of tyrosine and histidine side chains in proton transfer in catalysis we explored the dependence of the rate of cleavage of the splayed Y-junction by hANKLE1_331–615_R492H upon pH. Cleavage rate was measured in the presence of Mn^2+^ ions over the range 5.5–7.8 (Figure 5B). The rate rises over the pH range 5.5–7.2, well fitted by a single ionization corresponding to a p*K*_a_ = 6.9 (Figure 5C). As pH values rise above 7.2 the rate falls faster than can be fitted by a second ionization. The pH dependence of the rate of cleavage by hANKLE1_331–615_R492H H498A was also measured and the data plotted in Figure 5B and C. These data exhibit a different profile, with a steady increase in cleavage rate across the whole range of pH. The measured value of the p*K*_a_ coupled with the effect of the H498A mutation on both rate and pH profile indicates that histidine 498 likely participates in proton transfer in the reaction mechanism for phosphodiester hydrolysis.

### The dependence of ANKLE1_331–615_R492H cleavage rate upon temperature, and possible opening of the junction

**Figure 6.**
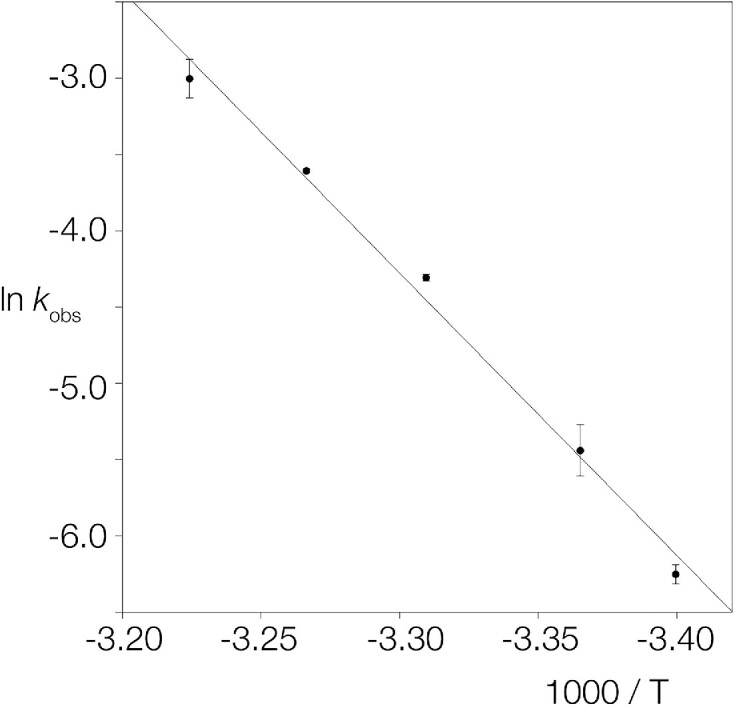
The temperature dependence the rate of cleavage of splayed Y-junction DNA by hANKLE1_331–615_R492H. Rates of cleavage were measured under standard conditions, initiated by addition of 10 mM MnCl_2_. The data have been plotted in the form of an Arrhenius plot, i.e. ln *k*_obs_ versus 1000/*T* where *k*_obs_ is the measured rate constant and *T* the absolute temperature. The rates (closed circles) were measured in triplicate, and standard deviations plotted. The line shows a fit to the Arrhenius equation, from which an activation energy *E*_a_ = 36.8 kcal mol^−1^ was calculated.

The progressive nature of the cleavage of DNA junctions by ANKLE1 suggests an opening of the dsDNA that progresses into the DNA with successive cleavage events. This might be reflected in the energy of activation for cleavage, and we therefore investigated the temperature dependence of the cleavage of the splayed Y-junction by hANKLE1_331–615_R492H in the presence of Mn^2+^ ions. The data are presented in the form of an Arrhenius plot of the natural logarithm of the observed cleavage rate (*k*_obs_) as a function of inverse temperature in Figure 6. The plot is linear, with a gradient corresponding to an activation energy *E*_a_ = 36.8 kcal mol^−1^.

We have also measured the rate of cleavage of a supercoil-stabilized cruciform as a function of temperature (Supplementary Figure S4). The Arrhenius plot is linear, with a gradient corresponding to an activation energy *E*_a_ = 36.9 kcal mol^−1^. This is almost the same as the value measured for the splayed Y-junction.

This relatively large activation energy would be consistent with a requirement for structural distortion of the DNA substrate concurrent with bond cleavage. One possibility would be an opening of the DNA helix. We explored whether any unpairing of the helix of the double-stranded portion of the splayed Y-junction DNA prior to cleavage could be detected using probing by potassium permanganate. Unstacked thymine nucleobases can be oxidised at the C5–C6 double bond to form *cis*-5,6-dihydroxy thymine that renders the backbone susceptible to cleavage by 1 M piperidine at 95°C. Splayed Y-junction was radioactively [5′-^32^P]-labelled on either the x-strand (the 5′ terminus of the duplex) or the r-strand (the 5′ terminus of the single-stranded flap) and incubated with no protein, or with 20 nM, 200 nM or 2 μM ANKLE1_331–615_ R492H in the presence of 1 mM EDTA, or ANKLE1_331–615_ R492H Y453F in the presence of 10 mM MgCl_2_ (Supplementary Figure S5). The reactivity of the single-stranded DNA is clear, and the double-stranded sections are much more protected. This pattern does not change with the addition of any concentration of protein. Thus these results do not support an opening of the DNA helix by hANKLE1_331–615_R492H in the absence of DNA cleavage.

## DISCUSSION

We have expressed the C-terminal fragment of human ANKLE1 comprising amino acids 331–615 in *E. coli* and found that it is active on splayed Y-junction junctions and supercoil-stabilized cruciform four-way DNA junctions with rates comparable to full-length ANKLE1 expressed in insect cells (8). A branchpoint is essential for ANKLE1 cleavage of DNA (i.e. a duplex is a very poor substrate), but the length of either or both single-stranded sections is not critical. We show here that hANKLE1_331–615_R492H is predominantly monomeric in free solution, and binds to a splayed Y-junction as a monomer, with high affinity (*K*_d_ ∼ 1 nM). This is fully consistent with the demonstration that the enzyme cleaves the four-way junction of a supercoil-stabilized cruciform structure unilaterally. Thus although a four-way junction is a substrate for ANKLE1, the result is a junction containing a single cleaved strand rather than complete resolution. Given that the N-terminal region of hANKLE1 that is not present in the 331–615 fragment has ankyrin repeats this could mediate protein-protein interactions that might be important in its function. However, the observation that hANKLE1_331–615_R492H acts in monomeric form is consistent with the primary substrate being the splayed Y-junction or similar structure, and ANKLE1 is most active on that branchpoint. Many GIY-YIG nucleases act in monomeric form (16,27). It is interesting to note that SLX1 (another GIY-YIG nuclease) is directly involved in four-way junction resolution, but works in partnership with another nuclease MUS81 to achieve the two cleavages required for resolution (1,28–31).

The pattern of cleavages on a splayed Y-junction by full-length ANKLE1 and hANKLE1_331–615_R492H are essentially identical, and both are consistent with a progressive movement along the dsDNA from the junction with the single-stranded DNA. We show here that the cleavage reaction is strongly temperature dependent, corresponding to a large activation energy *E*_a_ = 37 kcal mol^−1^; such a large activation energy is likely to arise from distortion of the DNA structure. The activation energy is virtually identical for cleavage of both the splayed Y-junction and the four-way junction of a cruciform, so is unlikely to arise from distortion of the junction structure. Moreover we observe no reactivity of the dsDNA to permanganate on addition of hANKLE1_331–615_R492H in EDTA or hANKLE1_331–615_R492H Y453F in the presence of Mg^2+^ ions, so in the absence of DNA cleavage the enzyme does not appear to induce local DNA melting in the ground state. DNA distortion might therefore involve unwinding of the DNA that preserves base pairing and does not result in elevated reactivity to permanganate. Or ANKLE1 might open the DNA locally as it cleaves, so strand separation is part of the activation process. Having made the first cleavage the enzyme can then make a second opening and cleavage, and so moves progressively into the dsDNA section of the junction.

The structure of the active center of human ANKLE1 modelled using AlphaFold shows three antiparallel strands of β sheet, with the putative functional tyrosine residues on the N-terminal (Y486) and central (Y453) strands. These are flanked above and below by helices on which are displayed the key histidine H498, glutamate E551 and asparagine N565. This arrangement of protein structure and the disposition of critical side chains is typical of a number of GIY-YIG nucleases that have been determined structurally (11–16). The putative active residues of ANKLE1 are clustered with a radius of 4.3 Å, and all exhibit significantly impaired activity on mutation.

**Figure 7.**
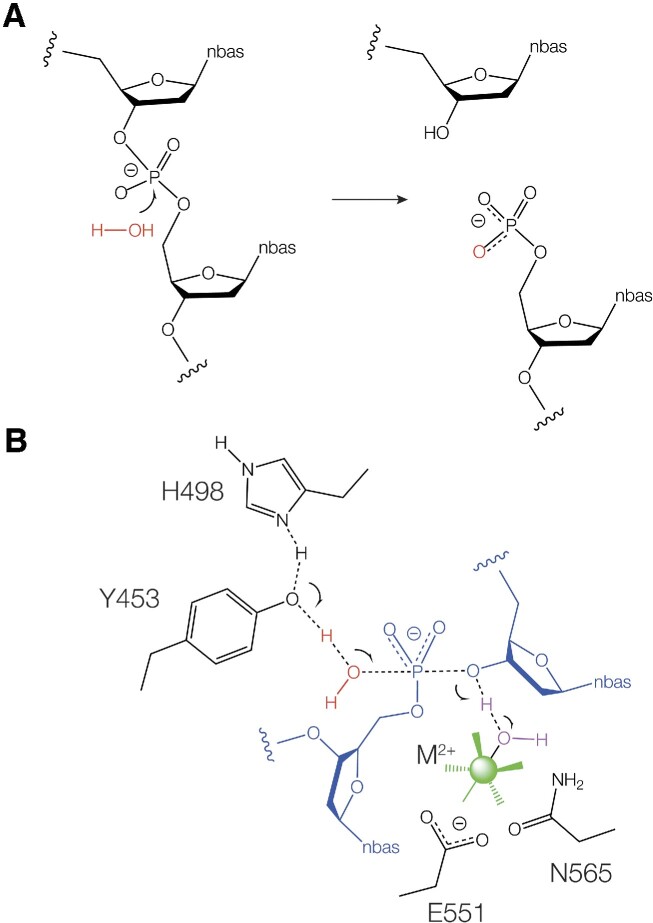
The proposed catalytic mechanism of phosphodiester bond hydrolysis by ANKLE1 consistent with the experimental data, and structural data on GIY-YIG nucleases in general. (**A**) The hydrolysis of a phosphodiester linkage in DNA by ANKLE1 leads to transfer of the phosphate to the 5′ end of the product DNA. (**B**) The transition state of the proposed catalytic mechanism showing general base catalysis by the combination of histidine 498 and tyrosine 453 in which a proton from the water nucleophile (red) is transferred to the phenolic oxygen of the tyrosine, and general acid catalysis by a water molecule (magenta) in the inner coordination sphere of the divalent metal ion (green). The metal ion is bound by the side chains of glutamate E551 and asparagine N565. The DNA is shown in blue.

Like the majority of junction-selective nucleases, ANKLE1 catalyses the hydrolysis of a specific phosphodiester linkage in a DNA junction, with transfer of the phosphate to the 5′-terminus that is created (Figure 7A). In principle this is likely to be catalysed by general acid-base catalysis in which the water nucleophile is deprotonated and the 3′-oxyanion leaving group is protonated. Many nucleases employ two-metal ion mechanisms in which metal ion-bound water molecules act as general base and general acid. However the GIY-YIG nucleases depart from this paradigm.

Our data suggest two important elements in the catalysis of phosphodiester bond cleavage by ANKLE1. First, atomic mutation of Y453 in which only the phenolic oxygen is removed leads to undetectable levels of activity. The reaction rate of the unmodified enzyme depends on pH over the range 5.5–7.0 corresponding to a p*K*_a_ = 6.9. That would be consistent with a histidine undergoing proton transfer in the transition state, and the pH profile of the H498A mutant is substantially altered. In the structure predicted by AlphaFold, N2 of H498 is 2.9 Å from the phenolic oxygen of Y453. A very similar arrangement of tyrosine and histidine was observed in the crystal structures of *Eco*29kI (13) and SLX1 (17) with histidine N2 2.6 and 2.7 Å from the oxygen of the tyrosine respectively. We therefore hypothesize that the proton from Y453 is transferred to H498, and the phenolic oxygen then acts as a general base to deprotonate the nucleophilic water molecule (Figure 7B). We propose that the second element in the catalysis by ANKLE1 involves a divalent metal ion. We have observed that the ANKLE1 reaction rate is log-linearly dependent on the p*K*_a_ of the ion present, implying that an ion-bound inner-sphere water molecule is involved in proton transfer in the transition state (Figure 7B). The ion is likely coordinated by E551 and N565; both E551A and N565D are totally inactive. A divalent metal ion was observed 2.1 Å from the carboxylate group of the corresponding glutamate in the crystal structure of UvrC (12) indicating inner-sphere coordination. Mutation of that glutamate, and also the corresponding one of SLX1 (15), led to complete loss of activity of both nucleases.

The general acid-base catalysis model we are proposing for human ANKLE1 is very similar to those proposed on the basis of crystallographic data for other GIY-YIG nucleases (12,13). However, kinetic ambiguity does not allow us to distinguish a model in which the tyrosine acts as general base and metal ion-bound water as the general acid or vice versa on the basis of our kinetic data. But comparison with crystallographic structures of other GIY-YIG nucleases bound to DNA shows that in general the critical tyrosine is observed to be on the side of the 5′O while the metal ion and the glutamate are found on the side of the 3′O (13,14,16). We therefore propose that in the hydrolysis of the phosphodiester linkage by ANKLE1 the tyrosine (assisted by the histidine) acts as the general base to deprotonate the water nucleophile, while the hydrated metal ion acts as general acid to protonate the O3′ leaving group as depicted in Figure 7B.

This model is consistent with all the available mechanistic data for ANKLE1, together with structural data for other GIY-YIG nucleases. Given the apparent similarities in active centers beween the GIY-YIG nucleases it is likely that they employ closely similar catalytic mechanisms, and that the mechanistic analysis described here can be extrapolated to the other enzymes. This might be of particular interest in the case of SLX1 that participates in four-way junction resolution in the context of the SLX1-SLX4-MUS81-EME1 complex (1,28,32–34), where it introduces one of the two required cleavage reactions and is thus comparable with ANKLE1 cleaving one strand in Y-shaped DNA junctions.

## DATA AVAILABILITY

The data underlying this article are available in the article and in its online supplementary material.

## NOTE ADDED IN PROOF

While this paper was in submission Chan and co-workers published a paper showing that human ANKLE1 is localized at the cell midbody: Jiang, H., Kong, N., Liu, Z., West, S. C. and Chan, Y. W. (2023) Human endonuclease ANKLE1 localizes at the midbody and processes chromatin bridges to prevent DNA damage and cGAS-STING activation. Adv. Sci. 10, e2204388.

## Supplementary Material

gkad416_Supplemental_File
