# Supplementary material for: Biochemical and mechanistic analysis of the cleavage of branched DNA by human ANKLE1
Source: Nucleic Acids Res. 2023 May 22;51(11):5743–54. doi: 10.1093/nar/gkad416 (PMC10287932; doi:10.1093/nar/gkad416)
Supplement: gkad416_Supplemental_File [file gkad416_supplemental_file.pdf]

Biochemical and mechanistic analysis of the cleavage of branched DNA by human ANKLE1

Alasdair D.J. Freeman, Anne-Cécile Déclais, Timothy J. Wilson and David M. J. Lilley

Supplementary Information

Supplementary Figures

MSPILGYWKIKGLVQPTRLLLEYLEEKYEEHLYERDEGDKWRNKKFELGLEFPNLPYYIDGDVKLTQS
MAIIRYIADKHNMLGGCPKERAEISMLEGAVLDIRYGVSRIAYSKDFETLKVDFLSKLPEMLKMFEDR
LCHKTYLNGDHVTHPDFMLYDALDVVLYMDPMCLDAFPKLVCFKKRIEAIPQIDKYLKSSKYIAWPLQ
GWQATFGGGDHPPKSDLEVLFQGPLGSM_{331}ATLWLTEDEASSTGGREPVGPCRHLPVSTVSDLELLKG
LRALGENPHPITPFTRQLYHQQLEEAQIAPGPEFSGHSLELAAALRTGCIPDVQADEDALAQQFEQPD
PARRWREGVVKSSFTY_{453}LLLDPRETQDLPARAFSLTPAERLQTFIRAIFY_{486}VGKGTH_{492}ARPYVH_{498}
LWEALGHHGRSRKQPHQACPK_{519}VRQILDIWASGCGVVSLHCFQHVVAVEAYTRE_{551}ACIVEALGIQT
LTN_{565}QKQGHCYGVVAGWPPARRRRLGVHLLHRALLVFLAEGERQLHPQDIQARG

Supplementary Figure S1 The full amino acid sequence of GST-hANKLE1$_{331-615}$ R492H fusion, with the GST sequence written in blue. The hANKLE1$_{331-615}$R492H N-terminal methionine, R492H mutation and the key active side residues are numbered and written in red.

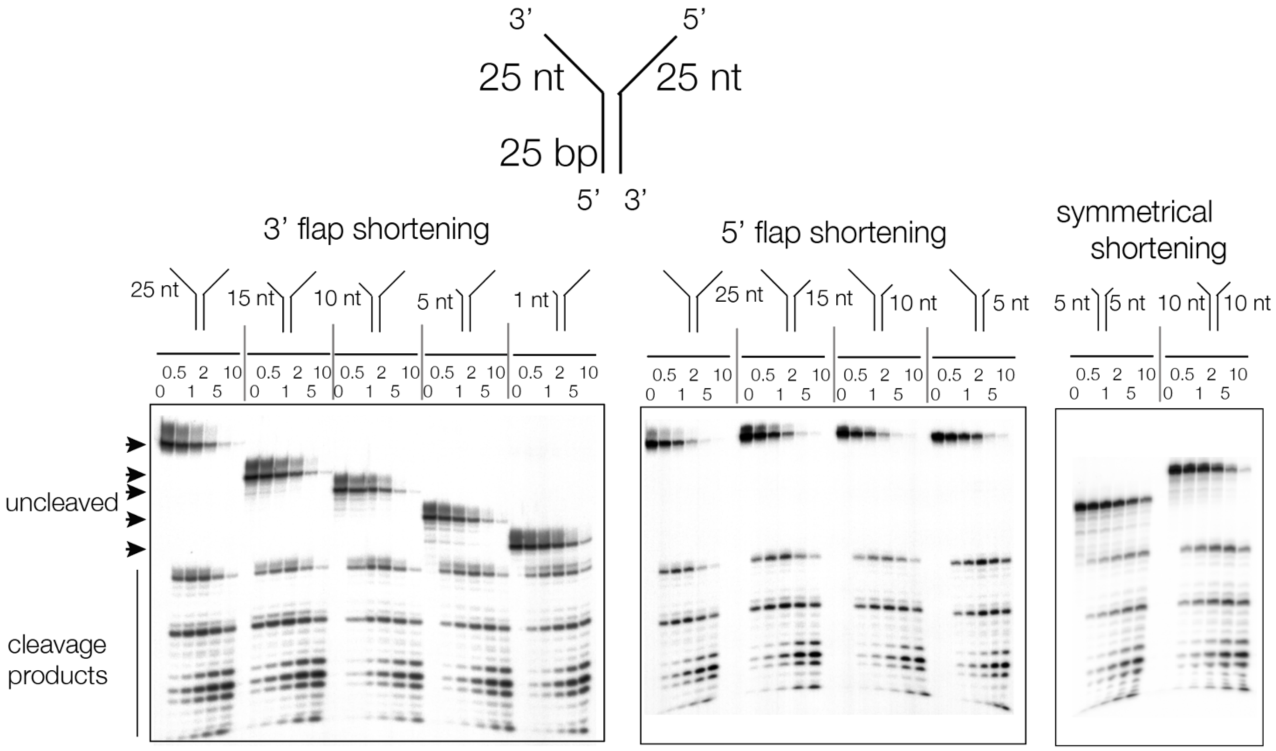

Supplementary Figure S2 Effect of flap length on cleavage of splayed Y-junction. A series of splayed-Y junctions with a double-stranded section of 25 bp and the 3' flap or the 5' flap or both were progressively shortened from 25 nt in length. Each was radioactively [5'-^{32}P]-labelled, and incubated with hANKLE1$_{331-615}$R492H under standard conditions. The products were resolved by electrophoresis in 15% (19:1) polyacrylamide, and dried before exposure to a storage phosphor screen.

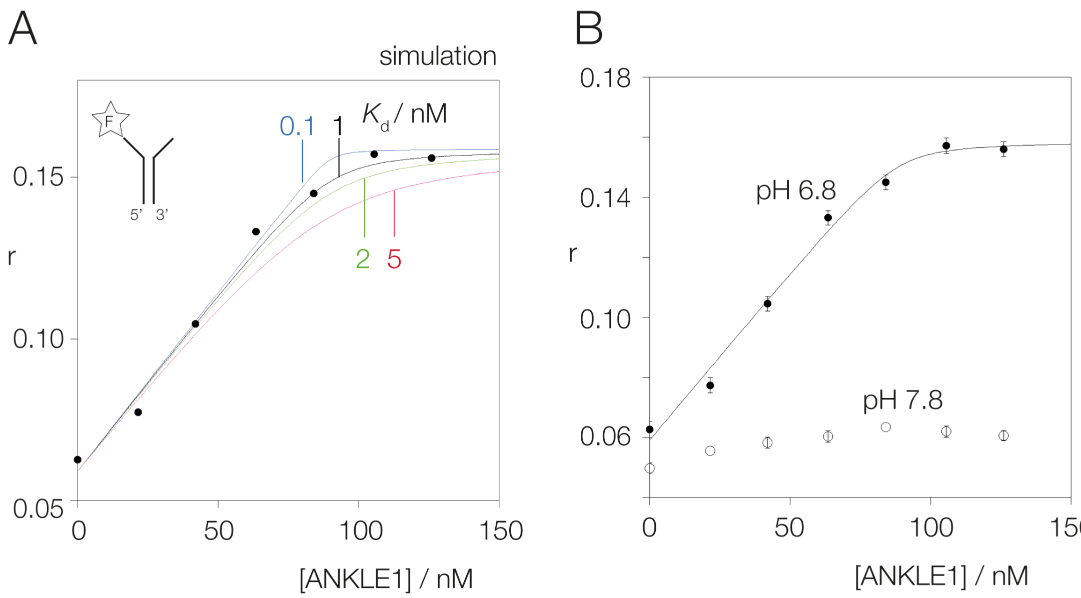

Supplementary Figure S3 Binding of hANKLE1$_{331\text{-}615}$R492H to a splayed Y-junction analysed by fluorescence anisotropy of fluorescein attached at the 5'-terminus of the single-stranded section. **A**. Simulation of binding isotherms using different values of binding affinity; K_d values used were 0.1 nM blue, 1 nM black, 2 nM green and 5 nM red. The experimental data are shown by the filled circles. The equation used to generate the simulations was :

$$r = r_0 + \Delta r.\frac{K_d + [Ank] + [Y] - ((K_d + [Ank] + [Y])^2 - 4[Ank].[Y])^{1/2}}{2.[Y]}$$

where r is the anisotropy at any given protein concentration, r_0 is the anisotropy in the absence of protein (using an empirical value of 0.059) and Δr is the change of anisotropy over the full range of the titration (using an empirical value of 0.0998). [Y] is the splayed Y-DNA concentration used in the titration, using an empirical value of 90 nM, and [Ank] is the concentration of hANKLE1$_{331\text{-}615}$ in nM. **B**. Fluorescence anisotropy (r) as a function of hANKLE1$_{331\text{-}615}$R492H concentration measured at pH 6.8 (closed circles) and pH 7.8 (open circles). The error bars indicate one standard deviation.

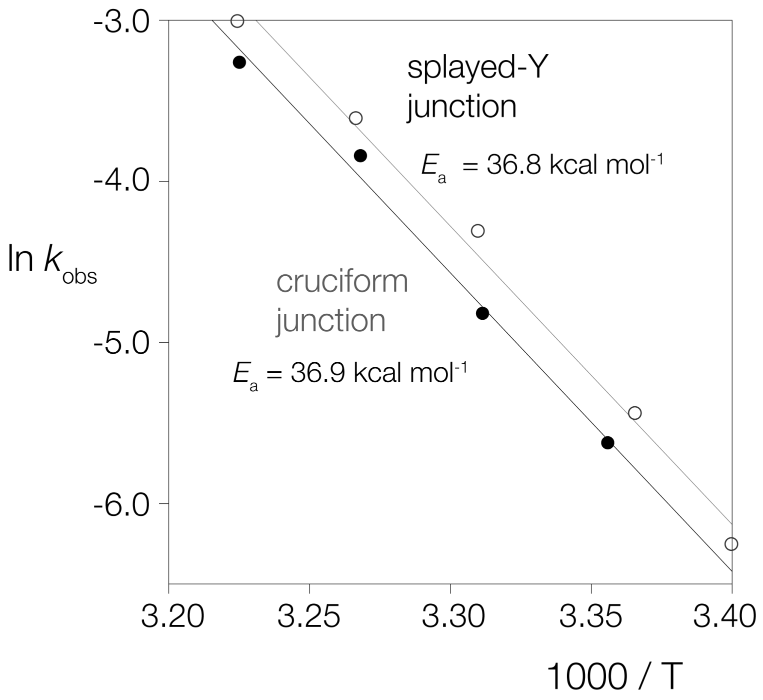

Supplementary Figure S4 Comparison of the temperature dependence of hANKLE1$_{331\text{-}615}$R492H cleavage of a splayed Y-junction and a supercoil-stabilized cruciform structure. Rates of cleavage (k_{obs}) of each substrate were measured under standard conditions in the presence of 10 mM Mn^{2+} ions (see Figures 3B and 6). lnk_{obs} has been plotted as a function of 1000/T, where T is the absolute temperature, and the data fitted to the Arrhenius equation :

$$k_{obs} = A \cdot e^{-E_a/RT}$$

where A is the pre-exponential term, R is the molar gas constant and E_a is the Arrhenius activation energy. Filled circles are the rates of cruciform cleavage while open circles are the rates of the splayed Y-junction. The Arrhenius activation energies are calculated from the product of the gradient with the molar gas constant. The data show that the activation energies for cleavage of the cruciform four-way junction and the splayed-Y junction in free junction are closely similar.

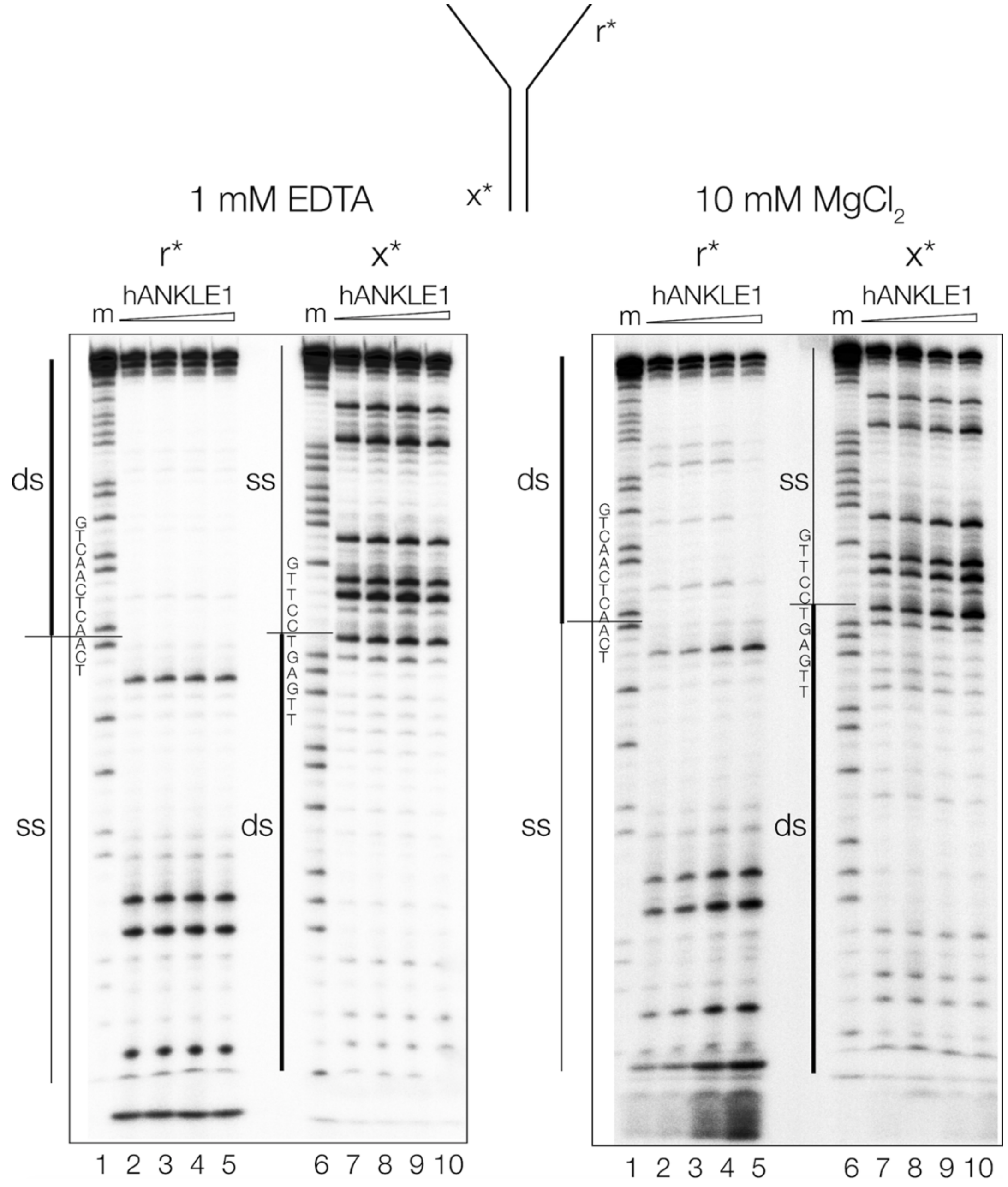

Supplementary Figure S5 Permanganate probing of thymine nucleotides in a splayed Y-junction. Splayed Y-junction DNA radioactively-[5'-^{32}P]-labelled on the indicated strand pre-incubated at room temperature for 5 min in the presence of 0 (tracks 2 & 7), 20 nM (tracks 3 & 8), 200 nM (tracks 4 & 9) or 2 μM (tracks 5 & 10) ANKLE1$_{331\text{-}615}$ R492H in 10 mM cacodylate (pH 6.5), 50 mM KCl, 50 μg/ml calf thymus DNA and either 1 mM EDTA or using ANKLE1$_{331\text{-}615}$ R492H Y453F 10 mM $MgCl_2$. After the reaction was terminated and the DNA precipitated, modified positions were cleaved by piperidine and the DNA analysed by electrophoresis in 15 % polyacrylamide under denaturing conditions. Tracks 1 and 6 contain purine sequence markers were generated by subjecting the same substrates reaction by formic acid followed by piperidine cleavage. The central sequences are shown on the left, with the junction between double and single stranded DNA indicated by the horizontal line.

Supplementary Table

Oligonucleotides used in these studies. All sequences written 5' to 3'.

Oligonucleotides used to make DNA substrates

h-strand: CATCTGTAAGCTTCCGGTAGCAGCCTGAGCGGTGGTTGAATTCACAGATG
r-strand *: CATCTGTGAATTCAACCACCGCTCAACTCAACTGCAGTCTAGAACACATG
x-strand: CATGTGTTCTAGACTGCAGTTGAGTCCTTGCTAGGACGGATCCCTCGAGG

* The r-strand oligonucleotide was also synthesized with a 3'fluorescein.

Mutagenic oligonucleotides

1. Oligonucleotides sequence for the deletion of the N-terminus of ANKLE1:
Forward: ATGGCAACCCTGTGGCTGAC
Back: GGATCCCAGGGGCCCCTG

2. Oligonucleotides for site directed mutagenesis:

Y453F	Forward: AGCTTTACCTTTCTGCTGTTAG<br>Back: GCTTTTAACAACACCTTC
Y486F	Forward: GCAATCTTCTTTGTTGGTAAAG<br>Back: ACGAATAAAGGTTTGCAG
H498A	Forward: TCCGTATGTTGCCCTGTGGGAAGC<br>Back: CGTGCATGGGTGCCTTTA
K519A	Forward: GGCATGTCCGGCAGTTCGTCAG<br>Back: TGATGCGGCTGTTTACGG
E551A	Forward: TATACCCGTGCAGCATGTATTG<br>Back: TGCTTCAACCGCAACAAC
N565D	Forward: GACACTGACCGATCAGAAACAG<br>Back: TGAATACCCAGGGCTTCA

The pHRX3 cruciform sequence within the *Eco*RI and *Bam*HI sites of pAT153

AATTCGCAGCCTGAGCGATATATATATATATATATATATATCGCTCAACTCAAG-3'
3'- GCGTCGGACTCGCTATATATATATATATATATATATAGCGAGTTGAGTTCCTAG

Supplementary Table S1 Sequence information. Sequences of oligonucleotides used in the construction of ANKLE1 substrates and for mutagenesis.
